# Real-World Use of Controlled Terminologies, Ontologies, and Vocabularies for Evidence Generation Across a Large International Observational Network: Challenges and Lessons Learned From a Mixed Method Study

**DOI:** 10.2196/92727

**Published:** 2026-09-15

**Authors:** Anna Ostropolets, Vlad Korsik, Tatsiana Skuhareuskaya, Aleh Zhuk, Maryia Khitrun, Alexander Davydov, Dmitry Dymshyts, Christian Reich, George Hripcsak, Patrick Ryan

**Affiliations:** 1 Observational Health Data Analytics, Johnson & Johnson, Raritan, NJ, 08869, United States; 2 Department of Biomedical Informatics, Columbia University Irving Medical Center, New York, NY, United States; 3 Odysseus, an EPAM Company, Cambridge, MA, United States; 4 Nemesis Health, New York, NY, United States; 5 Observational Health Data Analytics, Johnson&Johnson (United States), Titusville, NJ, United States

**Keywords:** ontology, terminology, interoperability, observational studies, real-world data, real-world evidence

## Abstract

**Background:**

Large-scale international real-world evidence generation benefits from terminology harmonization. Despite widespread adoption of standardized vocabularies, their effective use and long-term sustainability at scale remain poorly understood.

**Objective:**

This study aimed to examine real-world terminology and code use in data across a federated network of observational data sources.

**Methods:**

We conducted a 2-part survey of researchers and data owners within the Observational Health Data Sciences and Informatics community on their terminology use, challenges, and needs, accompanied by the analysis of code use across a subset of real-world data sources.

**Results:**

The survey covered 144 institutions across the United States, the United Kingdom, Europe, Asia, and Africa. Data on terminology use covered 60 sources, including 22 data sources that provided detailed code-level use information. We observed significant variations in terminology use, with 61 out of 89 terminologies used in the data present in less than 10% of the data sources. Code use even after data harmonization was also highly variable: less than 1% (95/742,337) of codes were found in all data sources. Mapping and hierarchy completeness, terminology coverage, versioning, and terminology changes were among the most common challenges. We outlined several of our subsequent process improvements: community contribution and stewardship pipelines, metadata for relationships, and informatics tools for assessment of the impact of terminology change.

**Conclusions:**

Terminology and coding inconsistencies across observational data sources require a standardized terminology system. Such a system is complex and time-consuming and needs community contribution and informatics solutions for harmonization to be scalable and sustainable. Even with a common reference standard, high heterogeneity of terminology and code use across different observational data sources remains.

## Introduction

Retrospective observational research using real-world observational data can provide insights into patient care and treatment outcomes across broad, diverse populations that may not be fully represented in randomized clinical trials [1]. Observational data networks collect information from various real-world data sources, such as electronic health records (EHRs), administrative claims, and registries, and provide larger patient samples, which is particularly relevant for addressing complex clinical questions, including those related to rare diseases or new drugs [2]. If networks are international, they can also capture care for diverse patient populations. On the other hand, the effective use of such networks for evidence generation requires reconciliation of differences across disparate ontologies, terminologies, and coding schemas to code health care events in different countries and institutions [3]. Despite the long-standing use of medical terminologies, no studies have examined the real-world manifestation and use of ontologies in observational data sources at scale.

Differences in data collection and coding can be overcome by using Common Data Models (CDMs), data harmonization, and quality assurance processes. One approach, used in large networks, is to preserve source content and coding and reconcile differences at the analysis stage [4]. Another approach, adopted by the Observational Health Data Sciences and Informatics (OHDSI) collaborative, is to use interoperable ontologies at the data conversion stage to enable standardized use of research tools [5].

OHDSI encompasses 544 data sources harmonized to the Observational Medical Outcomes Partnership (OMOP) CDM from 54 countries [6], including EHRs, administrative claims, registries, biobanks, and other data sources. OMOP CDM relies on a mandatory reference standard—OHDSI Standardized Vocabularies. The system is a harmonized collection of ontologies, terminologies, and controlled vocabularies and is used by all data owners in the OHDSI network to code patient data in the data sources converted to OMOP CDM. The OHDSI Standardized Vocabularies aim to achieve (1) comprehensive coverage of clinical events in the network by hosting all relevant local terminologies and (2) interoperability by supporting crosswalks among terminologies within the Vocabularies [5,7]. They have one referent term or concept per semantic entity (called standard) and an equivalence relationship from other terms with the same meaning (nonstandard) to the standard. For example, data sources in the United States contain *International Classification of Diseases, Tenth Revision, Clinical Modification* (*ICD10CM*) diagnostic codes, the United Kingdom uses Read codes, Korea uses KCD7, and China uses *International Classification of Diseases, Tenth Revision, Chinese Version* (*ICD10CN*). Within OMOP CDM, they are mapped to the Systematized Nomenclature of Medicine Clinical Terms (SNOMED CT) so that all converted data sources have SNOMED CT codes in standard fields and source terminologies in source fields available for standardized analytics.

Despite the widespread adoption of OMOP CDM and its central reliance on the Standardized Vocabularies, there remains limited empirical research on how individual terminologies within the Vocabularies are used in practical research. Prior literature documented challenges in terminology systems, including incomplete domain coverage, inconsistencies in hierarchies, and redundancy [8-11]. However, few studies have examined how these issues manifest in real-world, large-scale, multi-institutional research environments. It is also unclear how effectively a centralized terminology system can support diverse clinical data sources or how often data users encounter gaps that require manual curation or workarounds.

This study aims to fill that gap by systematically analyzing the use of terminologies and codes across real-world data sources within a global federated network. We combine quantitative analysis of terminology content and use patterns with qualitative insights from expert interviews to identify both patterns of use and persistent operational challenges. The study aims to (1) characterize the scale, complexity, and limitations of terminology integration in practical observational research and (2) outline methodological, process, and informatics solutions that are required to maintain a common terminology standard across disparate data sources.

## Methods

### Overview

To assess the use of terminologies across the international network of observational data sources (hereon, data sources), the OHDSI Vocabulary team, which maintains the OHDSI Vocabularies, conducted a 6-month landscape assessment in 2023 that concurrently included (1) a 2-part survey distributed across the OHDSI community and (2) in-depth interviews with the members of the OHDSI community.

A survey was disseminated to the OHDSI community members through (1) the mailing list of individuals who had downloaded the OHDSI Vocabularies (>8500 people); (2) continuous announcements on the OHDSI Microsoft Teams vocabulary channel (>1400 members), forums, and community calls; and (3) outreach to the working group leads and individual community members.

The first part of the survey (Table S1 in Multimedia Appendix 1) targeted data owners and included questions about the data source to which they had access; terminologies, vocabularies, classifications, ontologies, and coding schemes used in their data; the frequency of data and Vocabularies refreshes; and the Vocabulary version in use. Hereon, the OHDSI Standardized Vocabularies system will be referred to as Vocabularies, and individual ontologies, terminologies, classifications, or vocabularies within the system as terminologies, regardless of the level of their formalization.

The second part of the landscape assessment survey (Table S1 in Multimedia Appendix 1) targeted data owners, data conversion specialists, researchers, and other users of the OHDSI Vocabularies. First, the Vocabulary team asked the responders to score their confidence in Vocabularies’ integrity on a 5-point Likert scale. Questions included the tasks they use Vocabularies for, perceived Vocabularies’ completeness, correctness, intuitiveness of use, Vocabularies-related issues, and areas for improvement. We then analyzed anonymized results of the survey obtained from the Vocabulary team using a combination of deductive and inductive thematic analysis. All challenges were identified and listed based on the responder input. The challenges were then grouped together to form themes, which were subsequently discussed and refined with a second investigator. Finally, themes were organized according to an adaptation of the existing data quality framework by Khan et al [12].

The Vocabulary team concurrently used the convenience sampling method to select OHDSI open-source package and tool maintainers and leads of the working groups to conduct open-ended interviews. The interviews sought an in-depth understanding of the common challenges in using the Vocabularies within the OHDSI tools (CohortDiagnostics, FeatureExtraction, Atlas, and DataQualityDashboard) and their impact on clinical research (all clinical research groups: oncology and genomic, phenotyping, ophthalmology, dentistry, health equity, imaging, and psychiatry workgroups). During 1-hour semistructured online interviews conducted in January and February 2023, members of the Vocabulary team asked the interviewees to identify vocabulary dependencies in tools and research and provide an in-depth description of recent challenges they had experienced or that had been reported to them. At the end of the interview, key themes, challenges, and barriers were verbally summarized and validated with the interviewees, documented, and used to enrich the themes identified from the survey.

In addition, we asked data owners to supply aggregated code use information for their data sources. We collected standard and source codes along with their prevalence from the main OMOP CDM version 5 tables (condition_occurrence, procedure_occurrence, drug_exposure, device_exposure, measurement, observation). The executable SQL code distributed to data owners is available on the study package GitHub page [13]. The output of the code comprised a list of concept identifiers from the OHDSI Standardized Vocabularies along with the associated frequency in the data. We aggregated the data across the data sources and analyzed and plotted the distribution of the unique and overlapping concepts.

Results of the landscape assessment informed the prioritization of systematic improvements to the content and processes of the Vocabularies. These improvements were initiated after the completion of the assessment and have continued from 2023 to the present. The Improvements section describes content improvement as well as process improvement (regular cadence, assessment of principles and changes, collaborative development, and community engagement) and their connection to the results of the assessment.

### Ethical Considerations

The landscape assessment was reviewed by the Columbia University Institutional Review Board and was determined not to require further review.

## Results

We first assessed real-world terminology and code use in data across the network.

### Vocabulary Use in Source Data

The landscape assessment provided information on Vocabularies’ use across 60 data sources. Most of them originated in North America (United States: n=26, 43%, and Canada: n=3, 5%) and Europe (Belgium: n=5, 8%; Spain, Finland, Germany, Italy, and the United Kingdom: n=3, 5% each; the Netherlands: n=2, 3%; Norway, Estonia, Denmark, and Bulgaria: n=1, 2% each). Additional data sources originated from the Asia-Pacific region (Korea: n=2, 3%; and India and Japan: n=1, 2% each), as well as South America (Colombia: n=1, 2%). Most of the data were EHRs from tertiary health care centers and primary care centers (n=41, 68%), followed by administrative claims databases (n=9, 15%), registries (n=4, 7%), biobanks (n=3, 5%), clinical trial data (n=1, 2%), surveillance data (n=1, 2%), and combined EHR-registry dataset (n=1, 2%).

We observed high variability in vocabulary use across the data sources. While there were 12 terminologies used in at least one-third of the data sources, 16 additional terminologies were used in less than one-third but more than 10% of the data sources (such as CVX for vaccine capture; 11/61, 18%), and 60 other terminologies were used in less than 10% of the data sources (Table S2 in Multimedia Appendix 1).

A total of 12 relatively common terminologies used in the data included the World Health Organization (WHO) and country-specific versions of the *International Classification of Diseases, Tenth Revision* (*ICD-10*) and *International Classification of Diseases, Ninth Revision* (*ICD-9*; n=50, 83% and n=44, 73%, respectively). Logical Observation Identifiers Names and Codes (LOINC), SNOMED CT, *ICD-9 Procedure Coding System* (*ICD9Proc*), and Current Procedural Terminology, Fourth Edition (CPT4) were used in more than half of the data sources, followed by the Anatomical Therapeutic Chemical (ATC) classification system, *ICD-10 Procedure Coding System (ICD-10-PCS*), Healthcare Common Procedure Coding System (HCPCS), RxNorm, National Drug Code (NDC), and *International Classification of Diseases for Oncology, Third Revision* (*ICDO3*) used in more than one-third of the data sources. *ICD9Proc*, CPT4, HCPCS, and *ICD-10-PCS* were used to code procedures and services; ATC, NDC, and RxNorm were used to code drugs; LOINC was used to code measurements and laboratory tests; and SNOMED CT was used to code for multiple domains. Use of SNOMED CT and LOINC included disparate local coding schemas (such as laboratory tests and flowsheets) that the data owners mapped to these terminologies.

### Code Use in Standardized Data

A subset of data sources also provided statistics on code use within their datasets. The aggregated dataset included code use data from 22 data sources, comprising 14 US and 8 non-US sources across administrative claims, EHRs, and hospital data (Table S3 in Multimedia Appendix 1), and comprised 742,337 unique standard concepts across 5 common OMOP CDM domains (Condition, Procedure, Measurement, Observation, and Drug). These data reflect code use following data standardization using the OHDSI Vocabularies.

**Figure 1.**
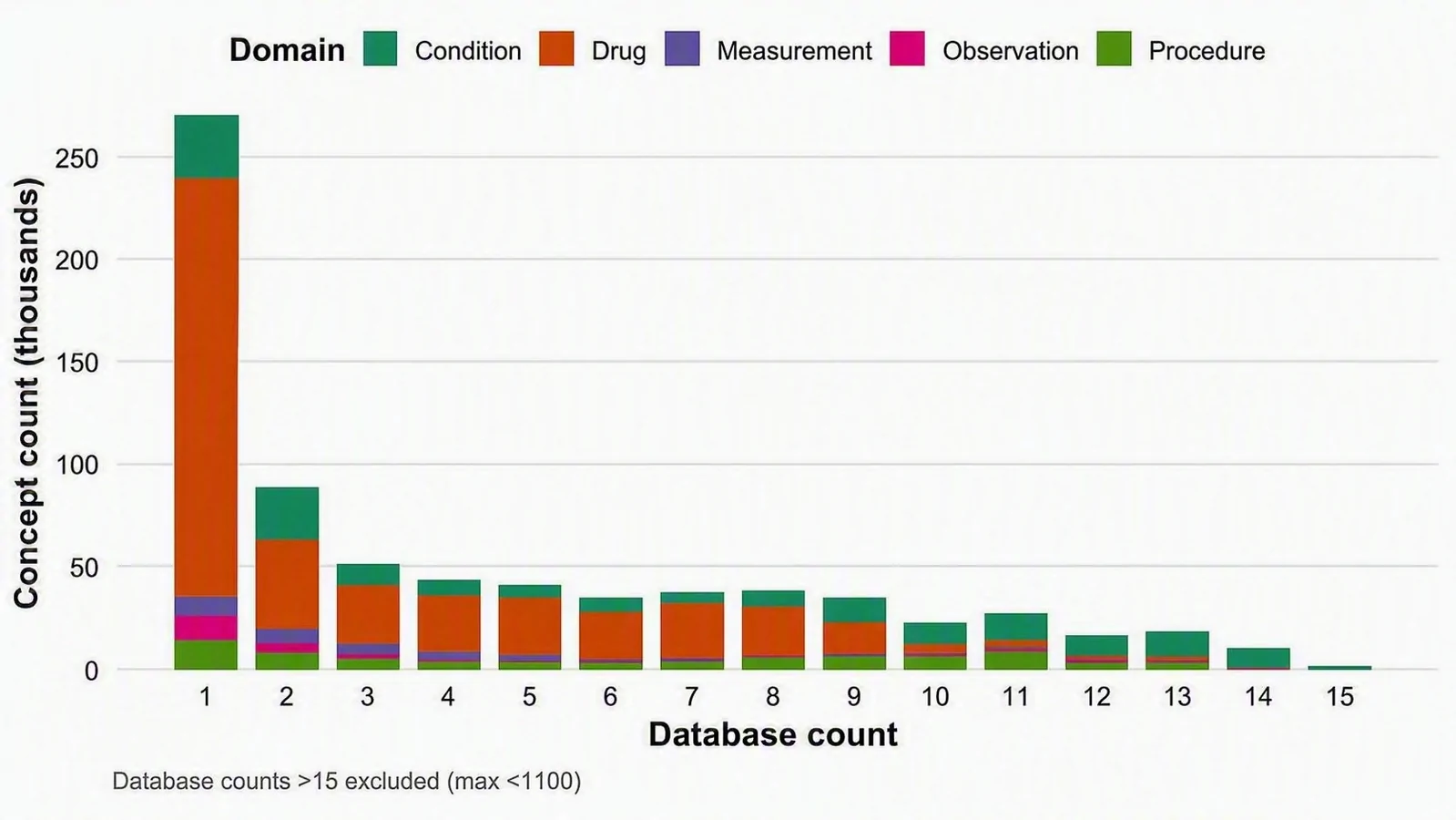
Distribution of concept counts across 22 data sources within the Observational Health Data Sciences and Informatics network, stratified by domain. The first bar represents the number of concepts found in 1 data source out of 22. Concepts found in more than 15 data sources (up to 1100) are not displayed for readability.

Despite data harmonization, substantial variation in code use remained across data sources in the OHDSI network: 36% (270,452/742,337) of concepts were unique to a data source, 11% (n=78,608) concepts were shared across at least half of the data sources, and less than 1% (n=95) of concepts were shared across all data sources (Figure 1).

Condition was the least heterogeneous domain, with the highest number of overlapping concepts across all domains, followed by procedures. Among all the domains, conditions underwent the longest and most extensive harmonization within the OHDSI Vocabularies. On the other hand, measurements lack established coding practices, explaining higher heterogeneity across domains. Drug coding variation could be due to different formulations in international markets.

**Figure 2.**
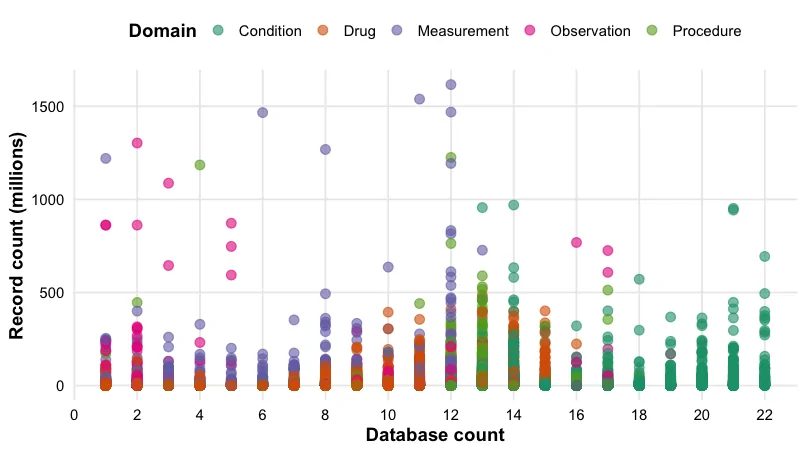
Distribution of patient record counts per code across 22 data sources within the Observational Health Data Sciences and Informatics (OHDSI) network, colored by domain. Each dot represents a code in the OHDSI Standardized Vocabularies.

Codes unique to a data source were not necessarily rare codes: some of the codes with a high number of patient records in data sources, such as laboratory tests, vitals, and scores, were only found in a few data sources (Figure 2).

Table 1 shows an example of standard codes commonly used across the network to code body temperature (with or without measurement results) after data harmonization efforts. One rare code had <2000 records across 8 data sources. On the contrary, some of the other more commonly used codes were found in 3 or fewer data sources: the most prevalent code “Temperature” was used only in 2 data sources, “Oral temperature” in 3 data sources, and “Temperature of skin” and “Temperature taking” in 1 data source.

**Table 1.** Selected measurement codes for body temperature measurement, with data source and patient record count across 22 data sources within the Observational Health Data Sciences and Informatics network.

Number of records	Number of databases	Concept name	Concept code	Vocabulary
1,302,569,491	2	Temperature	703421000	SNOMED CT^a^
34,433,988	9	Body temperature	8310‐5	LOINC^b^
13,105,216	3	Oral temperature	8331‐1	LOINC
4,168,603	12	Vital signs (temperature, pulse, respiratory rate, and blood pressure) documented and reviewed (CAP) (EM)	2010F	CPT4^c^
794,963	1	Temperature of skin	364537001	SNOMED CT
462,982	1	Temperature taking	56342008	SNOMED CT
1343	8	Monitoring of Temperature External Approach	4A1ZXKZ	*ICD-10-PCS* ^d^

a SNOMED CT: Systematized Nomenclature of Medicine Clinical Terms.

b LOINC: Logical Observation Identifiers Names and Codes.

c CPT4: Current Procedural Terminology, Fourth Edition.

d *ICD-10-PCS*: *International Classification of Diseases, Tenth Revision, Procedure Coding System.*

### Vocabularies’ Acceptance and Challenges

We then assessed how well Vocabularies facilitate data harmonization and standardized analytics across the network. The second part of the landscape assessment received 183 responses from community members from 144 institutions across the United States, the United Kingdom, Europe, Asia, and Africa. Overall, 87% (159/183) of the community were confident about the Vocabularies’ integrity, with more than one-quarter of responders (n=49, 27%) feeling extremely confident.

A substantial part of the community reported using the Vocabularies for more than 1 task. Of the 183 respondents, 143 (78%) use the Vocabularies for transforming data into the OMOP CDM; 119 (65%) use the Vocabularies for research (with characterization being the most common study type); and 51 (28%) use the Vocabularies for software, tools, and method development, including standardized analytics, data transformation, natural language processing, clinical decision support, and knowledge engineering.

On the basis of the results of the thematic analysis, community-identified challenges and areas for further development related to the Vocabularies were organized into 3 categories: conformance, completeness, and recency (Table 2) [12]. Within the conformance category, limited mapping completeness and multiple standard concepts per semantic meaning were the most reported problems that contributed to lower confidence in the Vocabularies’ integrity. Specifically, the lack of mappings for nonstandard source concepts was a particular issue for European data sources. While source codes were available for querying in OMOP CDM, the lack of mappings to their standard counterparts in the common reference standard limited the use of standardized OHDSI tools that rely on the latter.

**Table 2.** Main challenges with the Vocabularies and potential solutions.

Challenges	Example
Conformance	
Erroneous mappings from source to standard^a^	M08.00 “Unspecified juvenile rheumatoid arthritis of unspecified site” is now mapped to “Polyarticular juvenile idiopathic arthritis” but should be mapped to “Juvenile idiopathic arthritis”
Mappings associated with information loss^a^	Upward (uphill) mappings: Y82.8 “Other medical devices associated with adverse incidents” is mapped to “Medical accident to patient during surgical and medical care” One: many mappings: O33.7 “Maternal care for disproportion due to other fetal deformities” is mapped to “Fetal condition affecting obstetrical care of mother” and “Fetal disproportion”
Multiple standard concepts	SNOMED CT^b^ 23986001 Condition “Glaucoma” and UK Biobank 100523‐2 survey answer “Glaucoma” (question: “Eye problems/disorders”)
Completeness	
Insufficient mapping coverage	CDISC code C199995 “/100x FIELD” is not mapped
Lack of comprehensive hierarchies	*ICD-10-PCS*^c^ 06LY7ZC “Occlusion of Hemorrhoidal Plexus Via Natural or Artificial Opening” is not hierarchically related to CPT4 1007789 “Hemorrhoidectomy, internal, by ligation other than rubber band”
Source vocabularies not included in the Vocabularies	RadLex and radiology AGFA Impax are not incorporated into the Vocabularies
Recency and versioning	
Variability in release cadence	Variable number of releases per year based on community requests
Maintenance of one version of the Vocabularies available for download	Only the current version of the Vocabularies is available for download on [14]
Unpredicted impact of the Vocabularies’ changes on ETL^d^ processes, research, and OHDSI^e^ tools	Changes in the SNOMED CT hierarchy result in different sets of codes across different versions Changes in domain assignment across versions impact table assignment during ETL

a Source codes are available for querying in the Observational Medical Outcomes Partnership Common Data Model.

b SNOMED CT: Systematized Nomenclature of Medicine Clinical Terms.

c *ICD-10-PCS*: *International Classification of Diseases, Tenth Revision, Procedure Coding System.*

d ELT: extract, transform, and load.

e OHDSI: Observational Health Data Sciences and Informatics.

The presence of multiple standard concepts and the lack of comprehensive hierarchies posed challenges for defining sets of codes to identify patients of interest (phenotyping). A single semantic concept, such as a specific disease, may be represented by different codes in different countries and mapped to multiple standard concepts within the Vocabularies. Researchers reported difficulties identifying all relevant concepts within the Vocabularies to ensure that concept sets comprehensively represented the intended clinical concept and captured all eligible patients.

Within the completeness category, source terminologies not yet incorporated into the OHDSI Vocabularies were among the most reported problems. These terminologies could be classified into two categories: (1) public terminologies maintained by specialized authoring organizations and (2) unstructured elements present in the data as source coding schemes or free text. Public terminologies were European, such as local flavors of *ICD-10* or International Classification of Primary Care, which is associated with a recent uptake of newly harmonized data sources in the European Health Data and Evidence Network [15], as well as those covering new areas of interest in observational research (eg, RadLex and Radiology AGFA Impax for imaging data). Unstructured data source–specific elements not covered by the Vocabularies included poorly structured data elements from surveys and questionnaires, EHR flowsheets, and local codes used in registries.

Within the recency and versioning category, assessment of the impact of terminology changes on common research tasks, such as phenotyping, was one of the main challenges. Community members reported that changes across SNOMED CT hierarchy lead to changes in concept sets created on different versions of the Vocabularies. Similarly, differences in domain assignment across different versions resulted in the placement of the same codes in different OMOP CDM tables during data conversion, affecting their subsequent retrieval. This issue had not been notable prior to the landscape assessment, as its impact on study design and execution became apparent only as the number of studies using OMOP CDM increased and the effects of changes in source and standard vocabularies became more evident.

### Improvements

#### Overview

First, terminology use across the network informed the prioritization of terminologies for maintenance and improvement. This prioritization was undertaken by a newly established Vocabulary Committee, comprising diverse stakeholders from across the community and serving as a governance body. The process resulted in a roadmap and release schedule [16].

#### Recency and Versioning

Second, to address the observed variability in release cadence, we introduced a fixed semiannual cadence of releases, with releases in August and February every year aligned with the release cadence of source terminologies. The OHDSI Standardized Vocabularies had previously followed a flexible release cycle based on user requests and needs. It was hypothesized that semiannual releases would promote greater consistency in the Vocabularies’ version across the network, thereby facilitating more consistent research. Communication around releases was further formalized to make Vocabularies’ changes more predictable for the community. Specifically, advance notifications of upcoming changes [17] and detailed release notes [18] were introduced, incorporating plain language summaries of their potential impact on research and extract, transform, and load processes.

Semiannual releases enabled greater focus on content development addressing terminology gaps identified by the landscape assessment. For example, responders noted unstable domain assignment as disruptive to efficient phenotyping, which resulted in a dedicated release targeted at improving SNOMED CT processing and harmonization.

Additionally, unpredicted impact of Vocabularies’ changes was partially subsequently addressed by community-developed tools that assess the impact of Vocabularies’ changes on phenotyping [19] to allow the users to adjust cohort definitions based on changes in reference hierarchies and mappings and maintain concept sets consistent with the original logic.

Finally, to address the lack of versioning, we launched a versioning feature on Athena, OHDSI’s Vocabularies distribution service, in August 2024, which allows users to download different vocabulary versions and assess the delta between 2 given releases.

#### Completeness and Conformance

Besides focusing on the most used terminologies in the road map, we had to address the need for more terminologies and more mappings. Given that the demand substantially exceeded potential capacity of the road map, we developed a semiautomated pipeline to lower barriers to community engagement [20]. This pipeline included templates supporting the most commonly reported community use cases: seamless incorporation of new source terminologies into the Vocabularies, adding or changing mappings, and adding or changing concept attributes (community contribution GitHub page [20], part 1). These use cases could be handled through Microsoft Excel–based templates and were sufficiently covered by quality assurance checks to enable rapid integration into the subsequent vocabulary release cycle, thereby reducing the latency between identification of an issue and its resolution. Throughout the 4 releases in 2024 and 2025, we incorporated 17 contributions: 8 with mapping changes for 1 or more relationships, 4 with concept attribute changes, 3 deduplications of 1 or more standard concept pairs, and 2 with new source terminologies.

We also partially enabled more complex contributions, such as additions of complex standard terminologies, semantic deduplication, and hierarchy construction, although these activities require more complex, often expert-driven quality assurance. Over the past several releases, we processed 6 complex contributions, including updates to complex terminologies impacting reference sets, such as the Korean Electronic Data Interchange code system or the Columbia International eHealth Laboratory; addition of new drug terminologies; and development of new drug route hierarchies. The number of contributions grew over time and required extensive documentation on setting up a local instance of the Vocabularies’ schema so that contributors can perform terminology development, testing, and integration before contribution submission (community contribution GitHub page, part 2). Similarly, in response to the growing need for maintenance and improvement of existing terminologies, we started decentralization of terminology maintenance through the introduction of terminology stewards—individuals and institutions responsible for the maintenance of terminologies they use or are interested in.

Conformance challenges were addressed in several ways.

First, mapping errors have been addressed through targeted work on source terminologies within the semiannual release cycle, as well as enabling users to adjust mappings through community contributions.

Second, mapping with loss of information was addressed through introducing Simple Standard for Sharing Ontological Mappings (SSSOM) metadata for community contributions and for Vocabularies’ relationships. Metadata details include the mapping source, tool, confidence score, mapper, reviewer, and mapping predicate or precision. We curated SSSOM-compatible metadata for 142,113 mapping relationships [21]. Mapping precision has 3 categories: broadMatch (mapping with loss of granularity, 17% of the annotated mappings), narrowMatch (mapping with increased granularity, <1%), and exactMatch (83%). Metadata for concepts were also curated and distributed, including categories of nonstandard codes, as described in the Vocabularies’ interoperability documentation, and a reuse flag identifying codes reused across source terminologies [18]. Concept and relationship metadata were made publicly available and accompany each release [22].

Finally, together with the community, we have been addressing the challenge of multiple standard concepts. This challenge is most prominent in the areas where no single reference standard exists, and more terminology development work is warranted. Examples include harmonization of surveys and patient-reported outcomes, vaccine [23-25] and device [26] harmonization, development of ontologies to support observational research in Africa [27,28], and laboratory test harmonization. Complexity of harmonization pointed to the need for collaboration with other communities, such as the LOINC-SNOMED CT collaboration [29].

## Discussion

### Principal Findings

This work is the first study to examine real-world terminology use for observational research at scale. Our assessment of data sources from the United States, Europe, and the Asia-Pacific region revealed substantial heterogeneity of terminologies used in the data, with hundreds of terminologies, ontologies, and coding schemas used even within a limited subset of the data sources harmonized to OMOP CDM. Such diversity highlights the critical importance of ontology harmonization, as no single study protocol could feasibly account for the vast array of relevant codes across such a wide range of terminologies. Modern analytic techniques, such as the large-scale propensity score and predictive modeling, require far too many clinical variables as inputs to expect each data source owner to map all required source elements to concepts defined in a study. In this regard, the OMOP CDM, alongside the OHDSI Standardized Vocabularies, offers a unique advantage for generating generalizable and reproducible evidence by harmonizing both schema and content, as opposed to systems that rely solely on “adaptive rules” and keep source content inconsistent [4]. On the other hand, the approach of the common reference standard has been proven to be resource-consuming. OHDSI best practices require aligning terminologies to ensure a single standard concept per semantic meaning and constructing comprehensive hierarchies. As shown in the Results section, such an endeavor takes years and requires sustainable processes, community engagement, and collaboration with internal and external partners. These findings are consistent with the experience of other terminology organizations. For example, the SNOMED CT-LOINC collaboration, which focuses on harmonizing laboratory test concepts, was formally launched in 2022, with the first production release in 2025; harmonization efforts have continued since then.

Diversity in code use across the network also underscores a less-discussed challenge: the portability of phenotype or cohort definitions. When only a limited set of codes is shared across data sources, and the majority are unique to specific sources, a phenotype with high performance on one data source may not yield similar results on another. There is only limited research on patterns of phenotype portability [30-32], and borrowing phenotypes from the literature without adequate validation remains a common practice [33]. Although heterogeneity in coding practices has been highlighted before, researchers still rely on code sets developed in an institution to represent the same set of patients in their data [34]. Our findings further strengthen previous research and amplify the magnitude of observed heterogeneity across disparate data sources [35,36].

Beyond coding heterogeneity, changes in terminologies driven by an evolving understanding of diseases and quality improvements create additional challenges in ensuring temporal portability. A classic example of differences between *ICD-9* and *ICD-10*, which were reconciled through a CMS-curated crosswalk, is still being evaluated for validity in certain areas 8 years after the transition [37]. With dozens of terminologies in use in the network, harmonization becomes increasingly complex, as reflected in the results of the landscape assessment. The latter showed that erroneous mappings, mappings with loss of information, and imperfect hierarchies are among the common problems in terminology harmonization. These findings strengthen previous research showing persistent challenges in the alignment of commonly used terminologies [9-11,38,39]. Previous literature has mostly discussed the alignment of 2 given terminologies, whereas our experience highlights that the task becomes increasingly complex when aligning multiple terminologies.

We hypothesized that the solution to the scalability problem was to establish pipelines to enable people with different backgrounds from different countries to contribute to the Vocabularies’ content. Besides having domain expertise, we observed that community contributors had to learn about processes and operations within the OHDSI Vocabularies. Despite expanded documentation, tutorials, and other means of communication, the same questions continued to appear frequently in forums and working groups, suggesting that static documentation alone may not be sufficient to meet users’ learning needs. Previous research also highlights the importance of domain expertise in crowdsourcing [40] and suggests objective prequalification assessment as a means to improve collaborative authoring [41].

While our pipelines and documentation have supported community contributions, there remains significant potential for further development. New research domains within OHDSI—such as imaging, dentistry, and surveys—also necessitate both the addition of new content and the creation of conceptual models for content representation and quality assurance procedures to assess such representations, which delays implementation. Our experience with the contributions mirrors previously reported experience of other terminology systems, such as SNOMED, showing that contributions may introduce quality issues and require extensive quality assurance and curation [42,43], and points to the need for better informatics solutions. This observation is further supported by the growing body of literature examining the use of large language models and mixed approaches for terminology harmonization, including broad research and specific applications within OHDSI [44-48].

Overall, further work is needed to lower the barriers to community engagement and improve scalability and robustness, ensuring that a system of terminologies serving as the common reference standard can meet evidence-generation needs at scale.

### Limitations

This work has several limitations. First, as participation in the assessment was voluntary, not all the community members filled out the survey or contributed the data, which may limit the generalizability of the findings. Furthermore, because the total size and composition of the community are not precisely known, we are unable to reliably estimate the coverage of the survey responses. As such, the findings derived from survey data may reflect the perspectives of more engaged or vocal community members rather than the broader user base. Similarly, code use was assessed in the data sources that supplied information from 2 main regions, North America and Europe. Therefore, our findings may not be generalizable to other regions.

### Conclusions

While the use of a common reference standard, such as OHDSI Standardized Vocabularies, facilitates consistency in international observational research, substantial heterogeneity in terminology and code use across data sources remains even after harmonization. Efficient network research should account for such heterogeneity by developing tools and methods for robust phenotyping and making further efforts to improve Vocabularies’ coverage and quality. As the development and maintenance of such a system are complex, time-consuming endeavors, community input has played a central role in identifying high-priority content areas, surfacing usability issues, and revealing process inefficiencies. In parallel, content contributions from the community can be a sustainable approach to cover new use cases, increase the intake of new terminologies, and crowdsource improvement activities.

## Supplementary material

10.2196/92727 Multimedia Appendix 1 Observational Health Data Sciences and Informatics (OHDSI) Vocabularies’ landscape assessment survey questions and use of terminologies within the OHDSI Standardized Vocabularies in the data.

## Abbreviations

ATC: Anatomical Therapeutic Chemical
CDM: Common Data Model
CPT4: Current Procedural Terminology, 4th Edition
EHR: electronic health record
HCPCS: Healthcare Common Procedure Coding System
*ICD-10*: *International Classification of Diseases, Tenth Revision*
*ICD-10-PCS*: *International Classification of Diseases, Tenth Revision, Procedure Coding System*
*ICD-9*: *International Classification of Diseases, Ninth Revision*
*ICD10CM*: *International Classification of Diseases, Tenth Revision, Clinical Modification*
*ICD10CN*: *International Classification of Diseases, Tenth Revision, Chinese Version*
*ICD9Proc*: *International Classification of Diseases, Ninth Revision, Procedure Coding System*
*ICDO3*: *International Classification of Diseases for Oncology, Third Revision*
LOINC: Logical Observation Identifiers Names and Codes
NDC: National Drug Code
OHDSI: Observational Health Data Sciences and Informatics
OMOP: Observational Medical Outcomes Partnership
SNOMED CT: Systematized Nomenclature of Medicine Clinical Terms
SSSOM: Simple Standard for Sharing Ontological Mappings
WHO: World Health Organization
