# Supplementary material for: Real-World Use of Controlled Terminologies, Ontologies, and Vocabularies for Evidence Generation Across a Large International Observational Network: Challenges and Lessons Learned From a Mixed Method Study
Source: JMIR Med Inform. 2026 Sep 15;14:e92727. doi: 10.2196/92727 (PMC13577442; doi:10.2196/92727)
Supplement: Supplementary file 1 [file medinform-v14-e92727-s001.docx]

**Supplementary Table 1. OHDSI Vocabularies landscape assessment survey questions**

| **Question** | **Answer options** |
| --- | --- |
| *Part one: Vocabularies for ETL* | |
| Data source name | _____________________________ |
| OMOP CDM version | ☐ 3.0 ☐ 4.5 ☐ 5.3 ☐ 5.4 ☐ 6.0 ☐ Other: __________ |
| Current vocabulary version |  |
| What is the time difference between the last date observed in the source data and the date that ETL was executed? | _____________________________ |
| Frequency of data refresh | ☐ Daily ☐ Weekly ☐ Monthly ☐ Quarterly ☐ Semi-annually ☐ Annually ☐ Other: __________ |
| Frequency of vocabulary refresh | ☐ On each data refresh ☐ On each Vocabulary release ☐ Monthly ☐ Quarterly ☐ Semi-annually ☐ Annually ☐ Other: __________ |
| Which tables/fields populated with non-zero concepts from vocabularies | ☐ person.race_concept_id ☐ person.ethnicity_concept_id ☐ visit_occurrence.visit_type_concept_id ☐ condition_occurrence.condition_concept_id ☐ condition_occurrence.condition_status_concept_id ☐ drug_exposure.drug_concept_id ☐ drug_exposure.route_concept_id ☐ device_exposure.device_concept_id ☐ measurement.measurement_concept_id ☐ measurement.unit_concept_id ☐ procedure_occurrence.procedure_concept_id ☐ procedure_occurrence.modifier_concept_id☐ observation.observation_concept_id ☐ note.note_type_concept_id ☐ note.note_class_concept_id ☐ note.language_concept_id ☐ specimen.specimen_concept_id ☐ specimen.anatomic_site_concept_id ☐ specimen.disease_status_concept_id ☐ care_site.place_of_service_concept_id ☐ provider.specialty_concept_id ☐ payer_plan_period.payer_concept_id ☐ cost.cost_concept_id |
| Which specialty data elements do you have or plan to incorporate | ☐ Detailed cancer information (such as tumor stage) ☐ Structured genomic reports ☐ Imaging metadata ☐ Microbiology results ☐ None ☐ Other: __________ |
| Which OHDSI vocabularies are in your source data | ☐ ICD-9(CM) ☐ ICD-10(CM) ☐ ICD-9 (international) ☐ ICD-10 (international)  ☐ ABMS ☐ AMIS ☐ AMT ☐ APC ☐ ATC ☐ BDPM ☐ CAP ☐ CCAM ☐ CDT ☐ CGI ☐ CIEL ☐ CIM10 ☐ CIViC ☐ ClinVar ☐ CMS place of service ☐ CPT4 ☐ CTD ☐ CVX ☐ DA France ☐ dm+d ☐ DPD ☐ DRG (CMS) ☐ EDI ☐ Gemscript ☐ GGR (BSFI) ☐ GPI ☐ GGR ☐ HCPCS ☐ HemOnc ☐ HES Specialty ☐ HGNC ☐ ICD-10(CN) ☐ ICD-10(GM) ☐ ICD-10-PCS ☐ ICD-9-Proc ☐ ICD-9-Proc(CN) ☐ ICDO3 ☐ ISBT ☐ JAX ☐ JMDC ☐ KCD-7 ☐ KNHIS ☐ Korean Revenue Code ☐ LOINC ☐ LPD Australia ☐ LPD Belgium ☐ MDC (CMS) ☐ MedDRA ☐ Medicare Specialty ☐ MeSH ☐ MMI ☐ Multilex ☐ Multum ☐ NAACCR ☐ NCCD ☐ NCIt ☐ NDC ☐ NDFRT ☐ Nebraska Lexicon ☐ NFC ☐ NUCC ☐ OncoKB ☐ OncoTree ☐ OPCS4 ☐ OPS ☐ OXMIS ☐ PCORNet ☐ PHDSC ☐ PPI ☐ Read ☐ Revenue Code ☐ RxNorm ☐ SNOMED ☐ SNOMED Veterinary ☐ SOPT SPL ☐ SUS ☐ VA Product ☐ VANDF ☐ Other: __________ |
| Please list any source vocabularies, local coding schemes, or free text elements (such as ‘lab tests’) for which you use your own source-to-concept mapping or create custom concepts. |  |
| ***Part two*** | |
| What relational database management system(s) do you load the vocabularies into? | ☐ PostgreSQL ☐ MS SQL Server ☐ Oracle ☐ AWS Redshift ☐ Microsoft APS ☐ IBM Netezza ☐ Google BigQuery ☐ Apache Spark ☐ Databricks ☐ Apache Impala ☐ Other: __________ |
| When using ATHENA, did you run into issues with obtaining licensed vocabularies (such as not being able to have proof of license)? If so, please explain. | ____________________________________________________ |
| When using ATHENA, did you face technical issues downloading vocabularies (such as missing vocabularies)? If so, please explain. | ____________________________________________________ |
| After downloading vocabularies, did you face technical issues with uploading them to your local environment? If so, please explain. |  |
| Did you experience issues when migrating from one vocabulary version to another? If so, please explain. |  |
| Do you use the OHDSI Standardized Vocabularies to conduct clinical research? | ☐ Yes ☐ No |
| What type of studies are you involved in (check all that apply)? | ☐ Characterization: descriptive statistics, e.g., for natural disease history or treatment ☐ Population-level effect estimation: causal inference, e.g., for safety surveillance ☐ Patient-level prediction: ML for disease interception and precision medicine ☐ Other: __________ |
| Which vocabularies do you use in your studies (check all that apply)? | ☐ ATC ☐ ATC ☐  Cancer Modifer ☐ CIEL ☐ ClinVar ☐ CPT4 ☐ CVX ☐ EphMRA ATC ☐ HCPCS ☐ HemOnc ☐ HGNC ☐ ICD10PCS ☐ ICD9Proc ☐ ICDO3 ☐ MedDRA ☐ NAACCR ☐ Nebraska Lexicon ☐ OncoKB ☐ OncoTree ☐ OPCS4 ☐ RxNorm ☐ RxNorm Extension ☐ SNOMED ☐ SNOMED Veterinary ☐ UCUM ☐ VANDF ☐ Other: __________ |
| In your studies, have you ever used source (non-standard) concepts? If so, please explain why and provide examples. | ____________________________________________________ |
| In your studies, have you experienced challenges due to lack of ease or intuitiveness of the Vocabularies (such as not knowing how to use the ATC hierarchy)? If so, please explain. | ____________________________________________________ |
| In your studies, have you experienced challenges due to different vocabulary versions  (such as running a network study with two data partners with different vocabulary versions)? I  so, please explain. | ____________________________________________________ |
| Do you use the OHDSI Standardized Vocabularies to develop software? | ☐ Yes ☐ No |
| How do you use vocabularies in software development? | ____________________________________________________ |
| Do you use the OHDSI Standardized Vocabularies for any other tasks? If so, please clarify. | ____________________________________________________ |
| Evaluate the following statement:  I am conﬁdent in the integrity of the Vocabularies | ☐ Strongly agree ☐ Agree ☐ Disagree ☐ Strongly disagree |
| What would you need to see in the Vocabularies’ content or process to improve your conﬁdence? | ____________________________________________________ |
| Have your experienced challenges when attempting to use the Vocabularies documentation or educational materials? If so, please explain. | ____________________________________________________ |
| Have your experienced challenges with correctness of content of the Vocabularies (such as source concepts incorrectly mapped to standard, wrong domain assignment)? If so, please  explain. | ____________________________________________________ |
| Have your experienced challenges with completeness of the Vocabularies (such as source concepts not mapped to standards, missing source concepts, missing hierarchies, missing  vocabularies)? If so, please explain. | ____________________________________________________ |
| Have your experienced challenges with information loss/distortion due to source-to-standard mapping (such as the granularity of the mapping loosened part of the meaning of source concepts; multiple source concepts map to same standard)? If so, please explain. | ____________________________________________________ |
| Have your experienced challenges with recency of the Vocabularies (such as new source vocabulary version was released and new concepts appeared in your data but are not in the ofﬁcial OHDSI release)? If so, please explain. | ____________________________________________________ |
| Is there anything else you would like to share with us regarding your experience with the Vocabularies? | ____________________________________________________ |

**Supplementary Table 2.** Usage of the terminologies within the OHDSI Standardized Vocabularies in the data.

| Vocabulary name | Number of data sources, % | Vocabulary name | Number of data sources, % |
| --- | --- | --- | --- |
| ICD-10 (WHO, international versions) | 50, 83% | CAP | 3, 5% |
| ICD-9 (WHO, international versions) | 44, 73% | DPD | 3, 5% |
| LOINC | 41, 68% | EDI | 3, 5% |
| SNOMED | 34, 57% | CIViC | 3, 5% |
| ICD-9 Procedure | 32, 53% | HGNC | 3, 5% |
| CPT4 | 30, 50% | KCD-7 | 3, 5% |
| ATC | 27, 45% | OPS | 3, 5% |
| ICD-10 PCS | 26, 43% | OXMIS | 3, 5% |
| HCPCS | 25, 42% | PCORNet vocabulary | 3, 5% |
| RxNorm | 20, 33% | VANDF | 3, 5% |
| NDC | 20, 33% | CGI | 2, 3% |
| ICDO3 | 19, 32% | GGR (BSFI) | 2, 3% |
| CVX | 11, 18% | JAX | 2, 3% |
| NAACCR | 10, 17% | JMDC | 2, 3% |
| Medicare Specialty | 9, 15% | NFC | 2, 3% |
| Revenue Code (CMS) | 9, 15% | SOPT | 2, 3% |
| CMS Place of Service | 8, 13% | SNOMED Veterinary | 2, 3% |
| CDT | 7, 12% | AMIS | 1, 2% |
| DRG (CMS) | 7, 12% | AMT | 1, 2% |
| Multum | 7, 12% | BDPM | 1, 2% |
| NCIt | 7, 12% | Cancer Modifier | 1, 2% |
| NUCC | 7, 12% | CCAM | 1, 2% |
| ABMS | 6, 10% | CIEL | 1, 2% |
| dm+d | 6, 10% | DA France | 1, 2% |
| HemOnc | 6, 10% | Emphra ATC | 1, 2% |
| MedDRA | 6, 10% | GGR | 1, 2% |
| OncoKB | 6, 10% | GPI | 1, 2% |
| OncoTree | 6, 10% | KCD7 | 1, 2% |
| ClinVar | 5, 8% | ICMP2 | 1, 2% |
| MeSH | 5, 8% | KNHIS | 1, 2% |
| NDFRT | 5, 8% | LPD Australia | 1, 2% |
| Read | 5, 8% | LPD Belgium | 1, 2% |
| CTD | 4, 7% | MMI | 1, 2% |
| Gemscript | 4, 7% | Multilex | 1, 2% |
| HES Specialty | 4, 7% | NCCD | 1, 2% |
| ISBT | 4, 7% | OMOP Extension | 1, 2% |
| Korean Revenue Code | 4, 7% | OSM | 1, 2% |
| MDC (CMS) | 4, 7% | PHDSC | 1, 2% |
| Nebraska Lexicon | 4, 7% | Radiology AGFA Impax | 1, 2% |
| OPCS4 | 4, 7% | Radlex | 1, 2% |
| PPI | 4, 7% | SMQ | 1, 2% |
| SPL | 4, 7% | SUS | 1, 2% |
| VA Product | 4, 7% | UCUM | 1, 2% |
| APC | 4, 7% | UK Biobank | 1, 2% |

**Supplementary Table 3.** Data sources that contributed concept usage, the total number of condition records and unique condition concept codes per data source.

| **Database** | **Description** | **# of records** | **# of concepts** |
| --- | --- | --- | --- |
| IBM MarketScan Commercial Database (CCAE) | US commercial claims patients (0-65 years old) | 2,6 billion | 16,3 thousand |
| IBM MarketScan Multi-State Medicaid Database (MDCD) | Medicaid enrollees from multiple states | 1,3 billion | 16 thousand |
| IBM MarketScan Medicare Supplemental Database (MDCR) | Medicare supplemental coverage through privately insured, fee-for-service, point-of-service, or capitated health plans | 523 million | 15,6 thousand |
| Optum De-Identified Clinformatics Data-Mart-Database— Socio-Economic Status (SES) | Primarily representative of US commercial claims patients with socio-economic status information | 3 billion | 16,5 thousand |
| Optum De-Identified Clinformatics Data-Mart-Database—Date of Death (DOD) | Primarily representative of US commercial claims patients with full death record | 25 billion | 16,5 thousand |
| Optum De-identified Electronic Health Record Dataset (PANTHER) | Aggregated EHR repository from US health systems (Humedica’s EHR) | 956 million | 52,9 thousand |
| Premier Healthcare Database (PREMIER) | Hospital charge data from the hospitals across the US | 36 million | 16,2 thousand |
| The Healthcare Cost and Utilization ProjectNationwide Inpatient Sample (HCUP) | US hospital care data (inpatient stays, ambulatory surgery and services visits, and emergency department encounters.) | 1,6 billion | 9,6 thousand |
| IQVIA Longitudinal Patient Data (LPD)Australia | Longitudinal patient data from electronic health records from primary and secondary from Australian physician practices | 7,5 million | 3,5 thousand |
| IQVIA Disease Analyzer (DA) Germany | Electronic health records data from German practices (mostly primary care practices). | 339 million | 6,7 thousand |
| IQVIA Disease Analyzer (DA) France | Electronic health records data from French practices (mostly primary care practices). | 16,6 million | 3,4 thousand |
| Japan Medical Data Center database (JMDC) | Data from 60 society-managed health insurance plans covering workers aged 18 to 65 and their dependents in Japan | 101 thousand | 5,5 thousand |
| IQVIA US LRxDx Open Claims (Open Claims) | Anonymized, pre-adjudicated claims collected from US office based physicians and specialists | 6,2 billion | 16,6 thousand |
| IQVIA US Hospital Charge Data Master (CDM) | Anonymized hospital charge detail masters (CDM) collected from short-term, acute-care and non-federal hospitals | 5 billion | 16,2 thousand |
| IQVIA US Ambulatory EMR (AmbEMR) | EMR data from US primary care (40%) and speciality practices (60%) | 1,2 billion | 49,6 thousand |
| Stanford Medicine Research Data Repository (STaRR) | EHR data derived from outpatient and inpatient visits Stanford Hospital and Clinics | 545 thousand | 9 thousand |
| Korea National Health Insurance Service / National Sample Cohort (NHIS/NSC Korea) | National administrative claims database covering the South Korea population (2% population sample cohort from 2002 – 2013 | 5,4 billion | 6,8 thousand |
| Medical Information Mart for Intensive Care III (MIMIC3) | Electronic health records data associated with ~60,000 intensive care unit admission | 292 million | 0.8 thousand |
| Ajou University Database (Ajou) | Korean tertiary teaching hospital electronic health record data | 30,9 million | 6,3 thousand |
| Tufts Medical Center Database (Tufts) | Mixed EMR data including hospital data, state death records, tumor registry and primary and specialty practices | 124,4 million | 19 thousand |
| Australian Electronic practice based research network (AU-ePBRN) | Electronic health records data from primary care practices in Australia | 766 million | 2,8 thousand |
| Columbia University Medical Center Database (CUMC) | EHR data consisting of over 5 million patients from the New York-Presbyterian hospital and affiliated academic physician practice | 5,4 billion | 16 thousand |
